# Supplementary material for: Distinct effects of aging and klotho deletion on the choroid plexus
Source: GeroScience. 2026 Mar 25;48(3):3865–82. doi: 10.1007/s11357-026-02196-w (PMC13356169; doi:10.1007/s11357-026-02196-w)
Supplement: Supplementary file 1 — Supplementary file1 (PDF 2.03 MB) [file 11357_2026_2196_MOESM1_ESM.pdf]

Supplementary figures and tables Fanaei-Kahrani et al. Distinct Effects of Aging and Klotho Deletion on the Choroid Plexus

Supplementary Table 1. List of primers used for genotyping of newly born pups

Locus	Primer name	Primer sequence	Expected product size	PCR Program
**Slco1c1-Cre**	Alfi cre<br>Alfi cre rev<br>B2-1<br>B2-2	5'-AGA TGC CAG GAC ATC AGG AAC CTG-3'<br>5'-ATC AGC CAC ACC AGA CAC AGA GAT C-3'<br>5'-CAC CGG AGA ATG GGA AGC CGA A-3'<br>5'-TCC ACA CAG ATG GAG CGT CCA G-3'	+/+: 300bp<br>tg/+: 250bp + 300bp<br>tg/tg: 250bp	1. 94 °C - 3'<br>2. 94 °C - 30''<br>3. 60 °C - 30''<br>4. 72 °C - 1'<br>5. 72 °C - 10'<br>6. 10 °C - hold<br>Step 2.-4. 35x
**Klotho-flox**	Kl 33_fwd<br>Kl 34_rev	5'-TAT GTA GAG ?GG ?TT TAC AAG G-3'<br>5'-GTC TTC CCT GTG AAT CAG CC-3'	+/+: 195 bp<br>fl/+: 195+305bp<br>fl/fl: 305 bp	See above
**YFP**	oIMR4982 Mutant<br>oIMR8545 common<br>oIMR8546 wt	5'-AAG ACC GCG AAG AGT TTG TC-3'<br>5'-AAA GTC GCT CTG AGT TGT TAT-3'<br>5'-GGA GCG GGA GAA ATG GAT ATG-3'	+/+: 600bp<br>tg/tg: 320 bp<br>tg/+: 320+600 bp	See above

Supplementary Table 2. Reaction mix for genotyping of newly born pups

Component	for 20 µl reaction	Supplier
**5x GoTaq reaction buffer**	4 µl	Promega, M7911
**10x dNTPs**	2 µl	New England Biolabs, N0447S
**GoTaq polymerase**	0.1 µl	Promega, M7848
**Each primer**	0.5 µl	Eurofins Genomics
**Genomic DNA**	0.5 µl	
**ddH2O**	Up to 20 µl	

Supplementary Table 3. Differentially expressed proteins across different ages in FV-CP (Combined data from comparing Ctrl1 to $Kl^{\Delta CP}$ and Ctrl2 to $Kl^{\Delta CP}$)

Tissue/Age	number of proteins	Gene symbol		
FV-CP/M14-15, M2-3, M5-6	1	Abca5		
FV-CP/M2-3, M5-6	2	Ndufb9, Mgrn1		
FV-CP/M14-15, M5-6	1	TCAIM		

FV-CP/M2-3	35	*Mp68*<br>*Lifr*<br>*Banf1*<br>*Abcg2*<br>*Retsat*<br>*Jph1*<br>*Serpina3k*<br>*Slc7a1*<br>*Casp6*<br>*Sema7a*<br>*Dscr3*<br>*Hspb11*	*D2hgdh*<br>*Mepce*<br>*Mpdu1*<br>*Fuom*<br>*Sugp1*<br>*Ppl*<br>*Sfr1*<br>*Tmem27*<br>*Sphk1*<br>*C1qc*<br>*Arl8a*<br>*H3f3c; H3f3a*	*Dynlt1*<br>*Vtn*<br>*Rsrc2*<br>*Mog*<br>*Trappc4*<br>*Eps15*<br>*Adgrl1*<br>*Ceacam2*<br>*Tmem186*<br>*Atg12*<br>*Hrnr*
FV-CP/M5-6	53	*Coch*<br>*Fabp5*<br>*Atp5f1*<br>*Mettl13*<br>*Scpep1*<br>*Dpp4*<br>*ALB*<br>*Idh3g*<br>*Ptgds*<br>*Lsm3*<br>*Nptn*<br>*Ypel5*<br>*Wdr77*<br>*Fgfr1*<br>*Fam136a*<br>*Adam9*<br>*Taco1*<br>*Tmem263*	*Gstp1*<br>*Gstm5*<br>*Pcp4*<br>*Rab33b*<br>*Cfd*<br>*Wiz*<br>*Znf593*<br>*Sdhaf2*<br>*Cdkal1*<br>*Prr14*<br>*Ndufs8*<br>*Mcts1*<br>*Crabp2*<br>*Col6a1*<br>*Hspa14*<br>*Trmt6*<br>*Col6a2*<br>*Tmem209*	*Hspa1b; Hspa1a*<br>*Cox6a1*<br>*Atp8a2*<br>*Ifih1*<br>*Abi3*<br>*Sat2*<br>*Rpl38*<br>*Atp2a1*<br>*Atp1b3*<br>*Csnk2a1*<br>*Alg10b*<br>*Mrpl49*<br>*Tcp11l1*<br>*Aatf*<br>*Thap4*<br>*Mrps30*<br>*Nt5c3b*
FV-CP/M14-15	10	*Slc5a6*<br>*Nudt6*<br>*Atpaf1*<br>*HVM51*<br>*Emc6*	*Aldh3a1*<br>*Nfatc2*<br>*Znf207*<br>*Idua*<br>*Tubb1*	
FV-CP/M20-21	15	*Naaa*<br>*Baiap2l1*<br>*Gm11992*<br>*Nrm*<br>*Ifit3*<br>*Mrpl52*<br>*Rad23a*<br>*Mtnd5*	*Apob*<br>*Lamp2*<br>*Ubap1*<br>*Cfl2*<br>*Smad2*<br>*Efemp2*<br>*Zmat2*	

Supplementary Table 4. Differentially expressed proteins across different ages in LV-CP (Combined data from comparing Ctrl1 to $Kl^{\Delta CP}$ and Ctrl2 to $Kl^{\Delta CP}$)

Tissue/Age	number of proteins	Gene symbol	
LV-CP/M2-3, M5-6	1	*Sult1c2*	

LV-CP/M2-3	40	*Fbln5*<br>*Utp15*<br>*Lsm4*<br>*Cadm1*<br>*Osbpl3*<br>*Phf24*<br>*Thy1*<br>*Cnp*<br>*Fam241b*<br>*Gna11*<br>*Ptgr1*<br>*Aldh3a2*<br>*Igh-3*<br>*Mag*<br>*Thap4*<br>*Hat1*<br>*Dnajc5*<br>*Plcb1*<br>*Abca5*<br>*Nefl*	*Dnm2*<br>*Ndrg1*<br>*Cndp1*<br>*Alcam*<br>*Rbmx*<br>*Smap1*<br>*Cyp4v2*<br>*Hmgcl*<br>*Slc7a10*<br>*St3gal6*<br>*Srr*<br>*H2afy2*<br>*Ptprd*<br>*Rhbdf2*<br>*Ctnna2*<br>*C1qc*<br>*Fetub*<br>*Tmem70*<br>*Cpsf3l*<br>*Tbl1x*
LV-CP/M5-6	17	*Fuz*<br>*Ndel1*<br>*Sap30bp*<br>*Zfpl1*<br>*Ap3b2*<br>*Cln8*<br>*Gja1*<br>*Fabp7*<br>*Syt1*	*Nos1ap*<br>*Tspo*<br>*Camk2b*<br>*Il6st*<br>*Syn2*<br>*Ngly1*<br>*Tcp11l1*<br>*Phlda3*
LV-CP/M14-15	34	*Rpl35*<br>*Nme1*<br>*Sdpr*<br>*Csrp1*<br>*Tubb6*<br>*Hnrnpm*<br>*Fn3krp*<br>*Hist1h1d*<br>*Uqcrb*<br>*Tbc1d24*<br>*Alpl*<br>*Hn1l*<br>*Dysf*<br>*Chp1*<br>*Rwdd1*<br>*Mrc1*<br>*Cd63*	*Ddr1*<br>*Capza1*<br>*Steap3*<br>*Pcp4*<br>*Lamc3*<br>*Mavs*<br>*Fam103a1*<br>*Hdgf*<br>*Rps27l*<br>*Tubb2b*<br>*Arf5*<br>*Eef1a1*<br>*Arpc5*<br>*Ahcyl2*<br>*Rbmxl1*<br>*Pacrg*<br>*Rps10*
LV-CP/M20-21	5	*Col3a1*<br>*Fbn1*<br>*Arpp19*	*Col1a2*<br>*Pvalb*

Supplementary Table 5. KEGG pathway analysis of the proteins downregulated (in all ages) in $Kl^{\Delta CP}$ mice versus controls in the HC

Term	Adjusted P-value	Protein symbol
**Parkinson disease**	6.24E-04	Gnal; Ndufa4; Camk2a; Calm1; Tubb4a
**Long-term potentiation**	0.002421	Camk2a; Araf; Calm1
**Glioma**	0.002421	Camk2a; Araf; Calm1
**Pathways of neurodegeneration**	0.003417	Ndufa4; Camk2a; Araf; Calm1; Tubb4a
**Dopaminergic synapse**	0.007738	Gnal; Camk2a; Calm1
**Alzheimer disease**	0.010469	Ndufa4; Araf; Calm1; Tubb4a

Supplementary Table 6. KEGG pathway analysis of the proteins upregulated (in all ages) in $Kl^{\Delta CP}$ mice versus controls in the HC

Term	Adjusted P-value	Protein symbol
**ECM-receptor interaction**	1.80E-08	Col1a1; LAMA5; Lamb2; Col4a1; Lamc1; Hspg2
**Amoebiasis**	2.21E-08	Col1a1; Lama5; Gnal; Lamb2; Col4a1; Lamc1
**Focal adhesion**	2.69E-05	Col1a1; Lama5; Lamb2; Col4a1; Lamc1

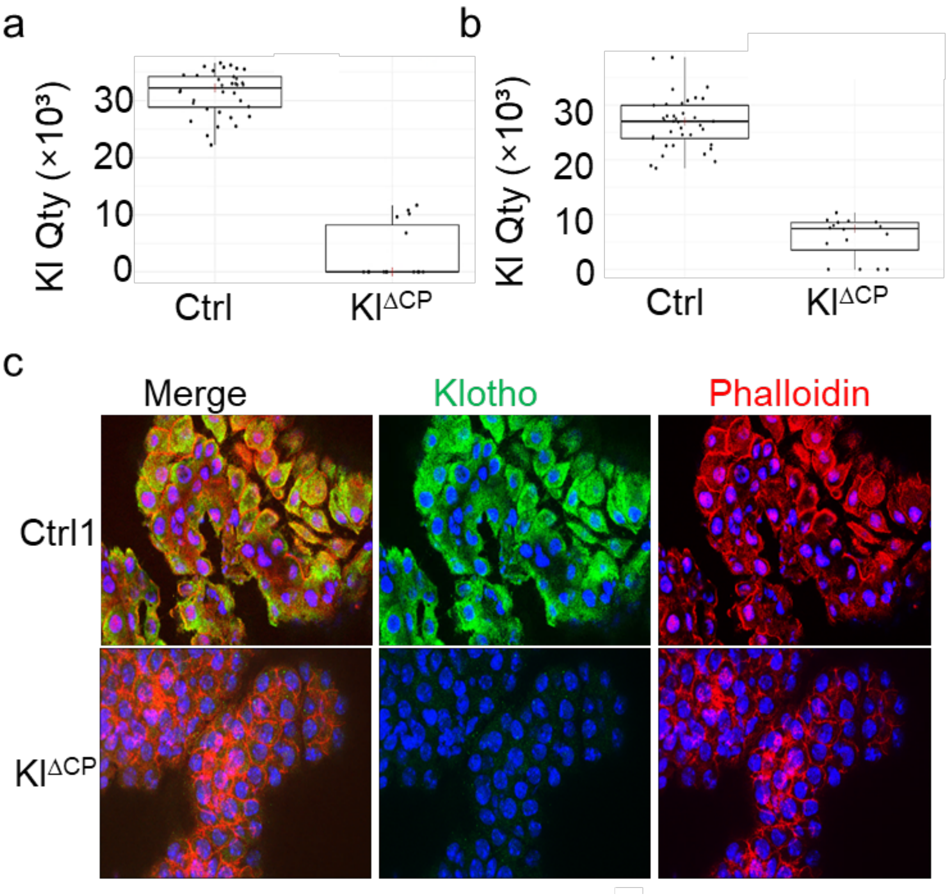

Supplementary Figure 1. Proteomics analysis and Immunofluorescent staining and confirmed the deletion of Kl in LV-CP of Kl$^{\Delta CP}$ mice. (a, b) Validation of Kl knockout in the CP of Kl$^{\Delta CP}$ mice using LC/MS proteomics of the FV-CP (A, n=36 controls, n=15 Kl$^{\Delta CP}$) and the LV-CP (B, n=37 controls, n=16 Kl$^{\Delta CP}$). (c) Brain sections from 2-3 months old Kl$^{\Delta CP}$ and control 1 mice were immunostained for Klotho and Phalloidin, and Z-stack images were acquired using the tile scan function on a Zeiss ApoTome microscope. Maximum intensity projections are shown. Nuclei were counterstained with Hoechst (blue). Scale bar = 20 µm.

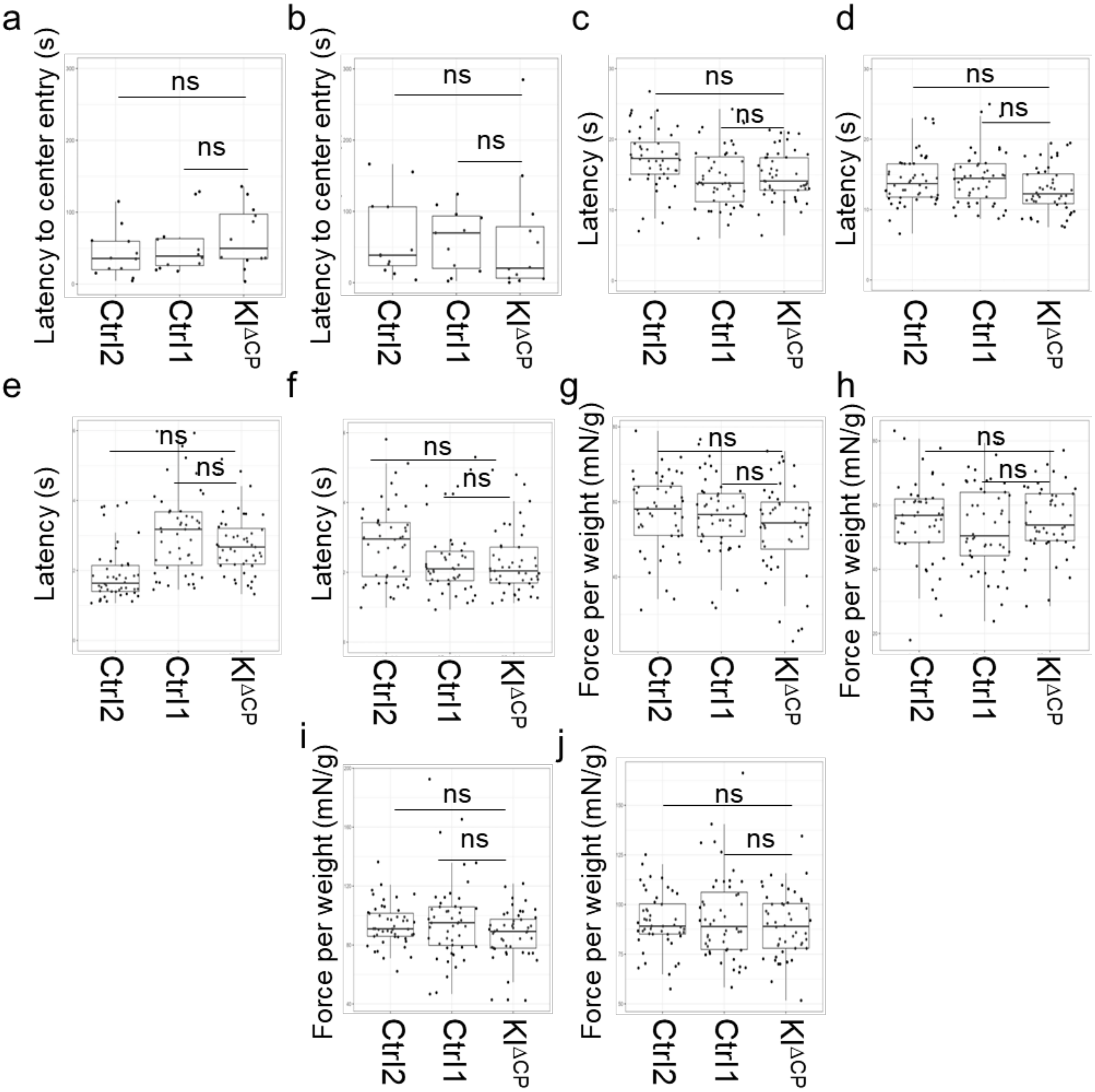

Supplementary Figure 2. Behavioral analysis of 2-5 months and 8-12 months KI$^{\Delta CP}$ mice compared to control groups showed no significant difference between them. (a, b) Open field test to measure exploration behavior (latency to center entry) between KI$^{\Delta CP}$ and control mice. The test involved placing the animal in a white plastic cube. Movement was tracked using a video system that was installed above the area. The latency to enter the center was assessed in (a) 2-5 months and (b) 8-12 months mice. (c, d) Hot plate test. Each mouse was placed on a 52°C preheated plate and the latency time until the occurrence of the first contact avoidance reaction was measured. Measurements were taken for (c) 2-5 months and (d) 8-12 months. (e, f) Tail flick response. In this experiment the mouse's tail of 2-5 months (e) and 8-12 months (f) was immersed in hot water at 45.0±0.2 °C and the reflexive tail movement time was recorded. (g-j) Grip strength

analysis measuring the peak force exerted by front paws or all four paws on a wire grid. Data represents (g) 2-paw strength in 2-5 months, (h) 2-paw strength in 8-12 months, (i) 4-paw strength in 2-5 months, and (j) 4-paw strength in 8-12 months. Statistical evaluation included RM-ANOVA and Cohen's d-effect size, with error bars representing standard deviation and each dot symbolizing a single mouse. Significance was set at p-value > 0.05 (ns).

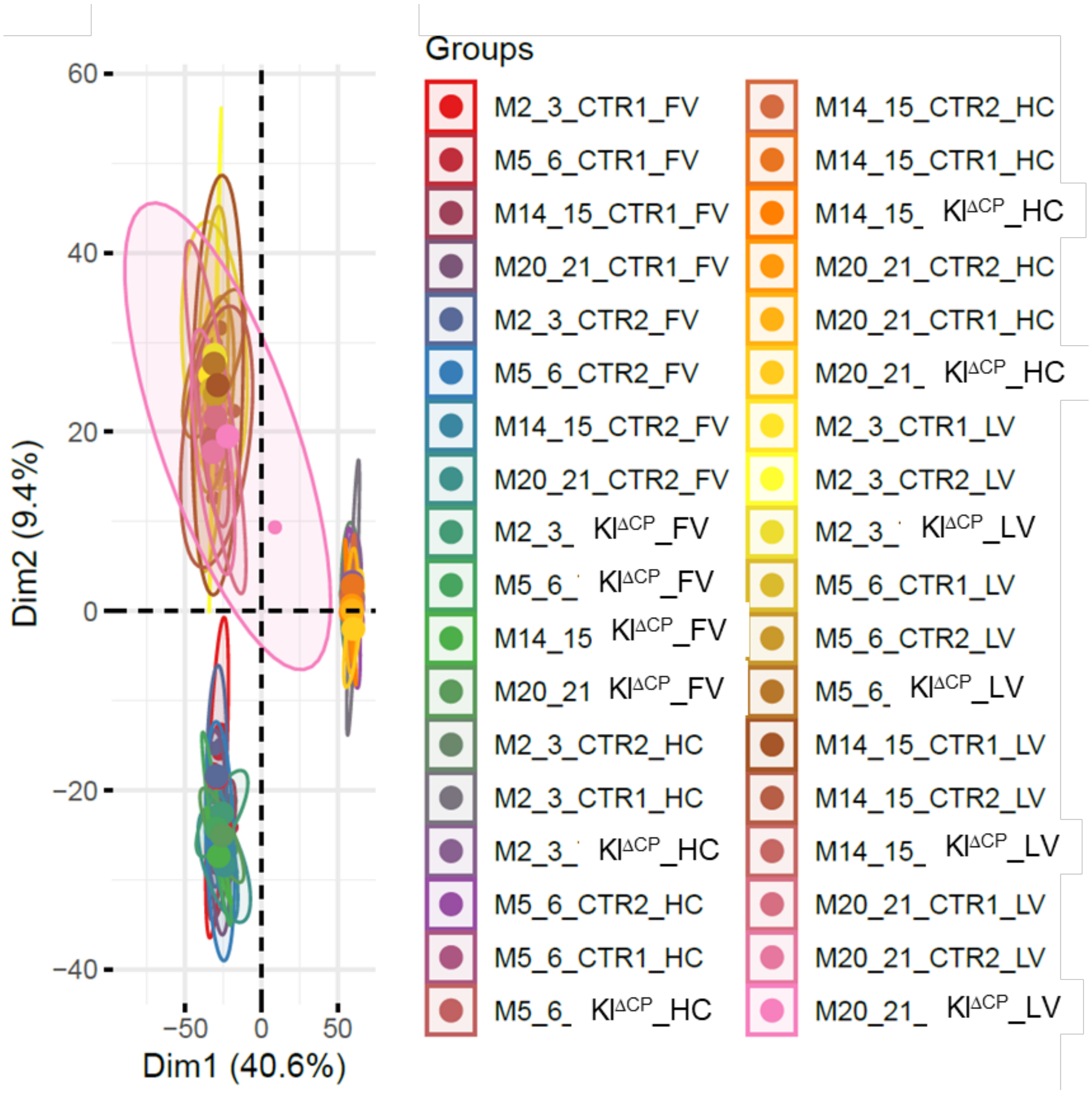

Supplementary Figure 3. Principal component analysis (PCA) illustratingthe distribution of all samples. The results show clear clustering of FV-CP, LV-CP, and HC samples within their respective groups, indicating reproducible replicate data and distinct proteomic profiles for FV-CP, LV-CP, and HC tissues (M: Month, CTR: Control, FV-CP: Fourth ventricle Choroid plexus, LV-CP: Lateral ventricle Choroid plexus, HC: Hippocampus).

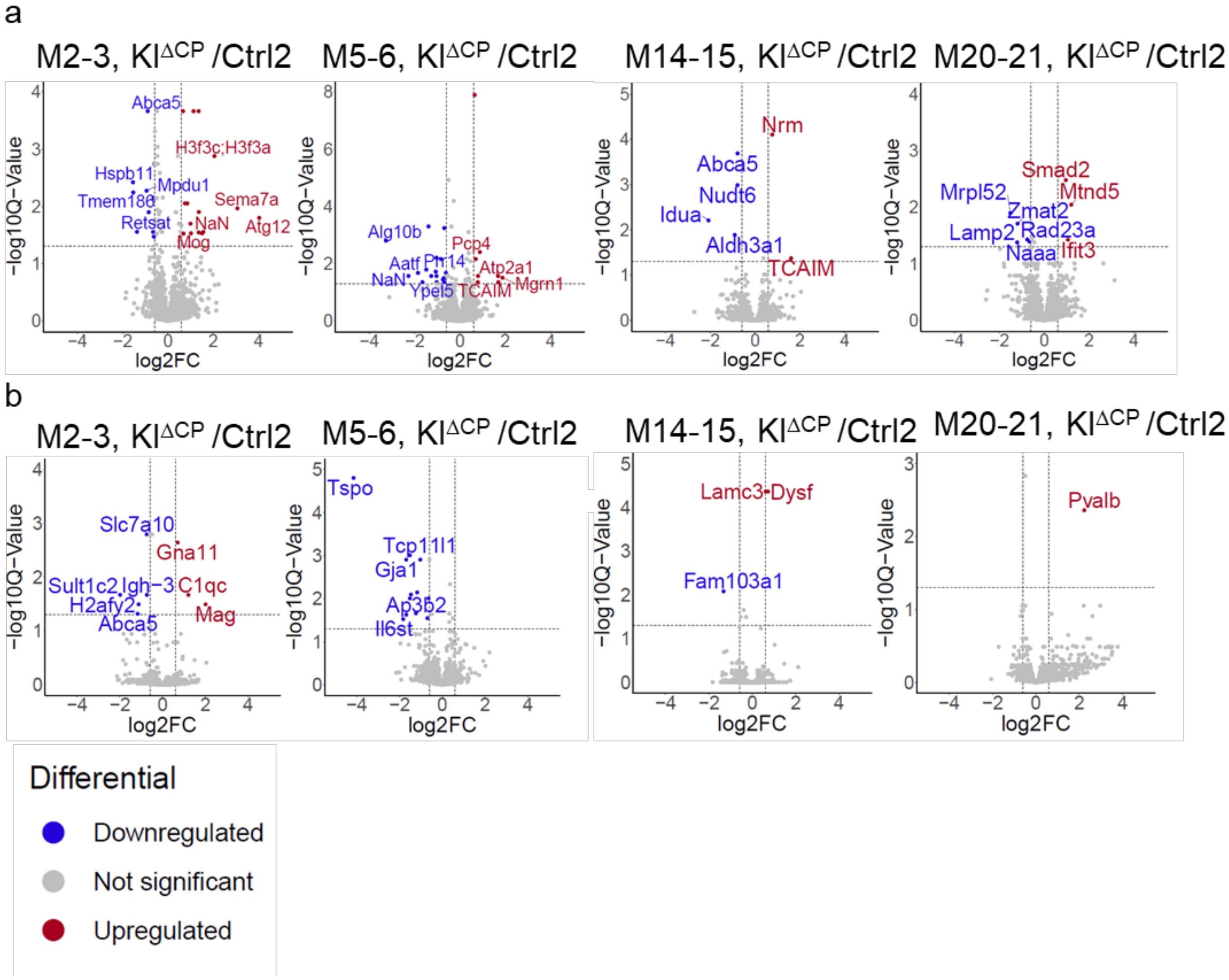

Supplementary Figure 4. Volcano plots depicting differential expression of proteins in the FV-CP (a) and LV-CP (b) in KI$^{\Delta CP}$ mice compared to Ctrl2 at various ages (2-3, 5-6, 14-15, and 20-21 months). Shown are protein symbols, the written names are the top 5 upregulated and downregulated proteins. Blue, significantly downregulated in KI$^{\Delta CP}$ compared to Ctrl2, red, upregulated in KI$^{\Delta CP}$ compared to Ctrl2.

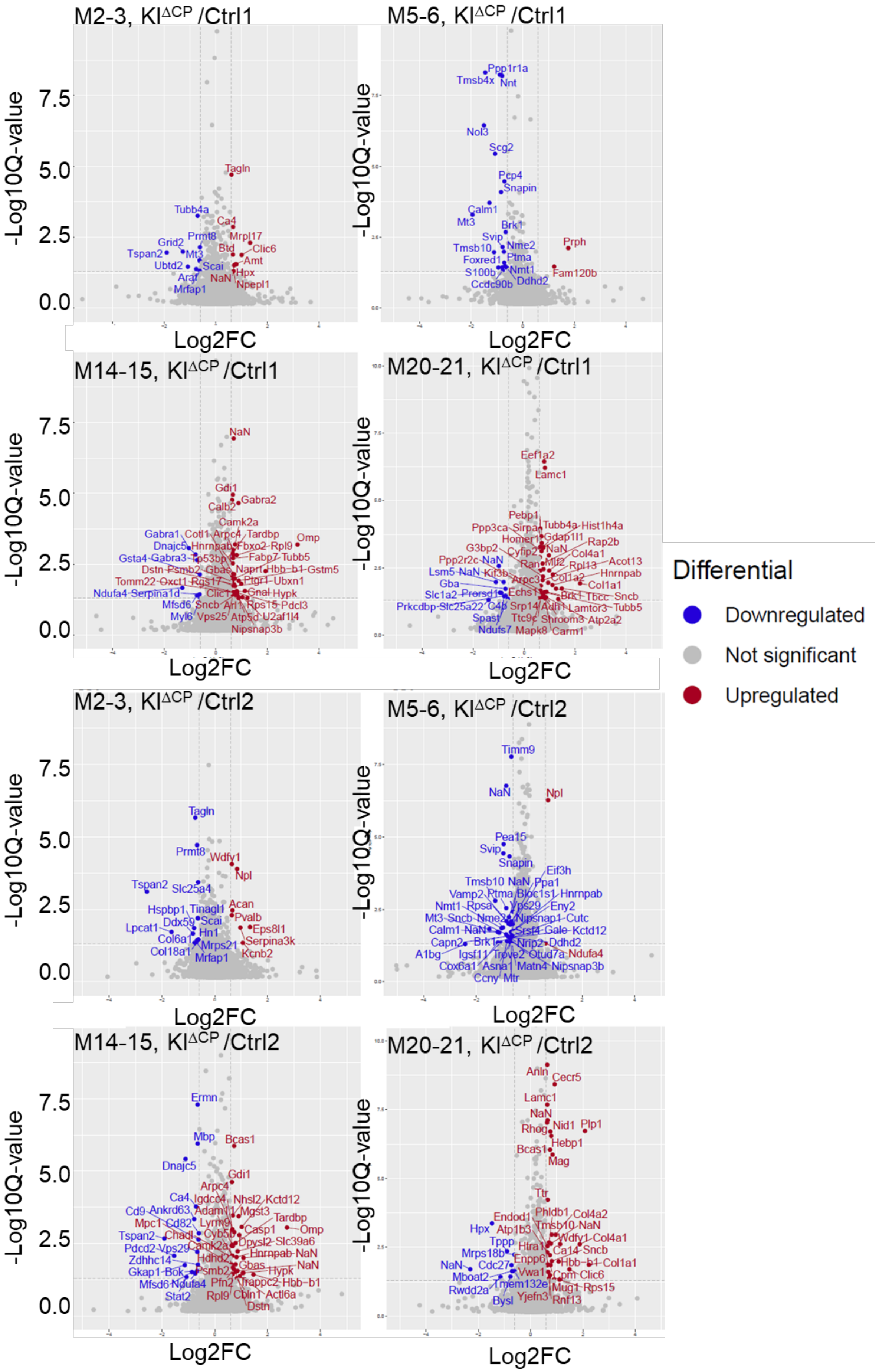
M2-3, KI$^{\Delta CP}$ /Ctrl1
-Log10Q-value
7.5
5.0
2.5
0.0
Log2FC
Tagln
Tubb4a
Ca4
Mrpl17
Prmt8
Grid2
Btd
Clic6
Tspan2
Mt3
Amt
Ubtd2
Scai
Hpx
Araf
NaN
Npepl1
Mrfap1
M5-6, KI$^{\Delta CP}$ /Ctrl1
Ppp1r1a
Tmsb4x
Nnt
Nol3
Scg2
Pcp4
Snapin
Calm1
Mt3
Brk1
Svip
Prph
Tmsb10
Nme2
Ptma
Foxred1
Nmt1
S100b
Fam120b
Ddhd2
Cdc90b
M14-15, KI$^{\Delta CP}$ /Ctrl1
NaN
Gdi1
Gabra2
Calb2
Camk2a
Gabra1
Cotl1
Arpc4
Tardbp
Omp
Dnajc5
Hnrnpab
Fbxo2
Rpl9
Gsta4
Gabra3
Tp53bp1
Fabp7
Tubb5
Dstn
Psmb2
Gbas
Naprt
Hbb-b1
Gstm5
Tomm22
Oxct1
Rgs17
Ptgr1
Ubxn1
Ndufa4
Serpina1d
Clic1
Gnal
Hypk
Mfsd6
Sncb
Arl1
Rps15
Pdcl3
Myl6
Vps25
Atp5d
U2af1l4
Nipsnap3b
M20-21, KI$^{\Delta CP}$ /Ctrl1
Eef1a2
Lamc1
Pebp1
Ppp3ca
Sirpa
Tubb4a
Hist1h4a
Homer1
Gdap1l1
G3bp2
Cyfip2
NaN
Rap2b
Col4a1
Ppp2r2c
NaN
Rang
Mlf2
Acot13
Lsm5
NaN
Kif3b
Rpl13
Hnrnpab
Gba
Arpc3
Col1a2
Col1a1
Slc1a2
Prorsd1
Echs1
Brk1
Tbcc
Sncb
Prkcdbp
Slc25a22
C4b
Srp14
Adh1
Lamtor3
Tubb5
Spast
Ttc9c
Shroom3
Atp2a2
Ndufs7
Mapk8
Carm1
Differential
Downregulated
Not significant
Upregulated
M2-3, KI$^{\Delta CP}$ /Ctrl2
Tagln
Prmt8
Wdfy1
Npl
Tspan2
Slc25a4
Acan
Hspbp1
Tinagl1
Pvalb
Ddx59
Scai
Lpcat1
Eps8l1
Hn1
Col6a1
Serpina3k
Mrps21
Col18a1
Kcnb2
Mrfap1
M5-6, KI$^{\Delta CP}$ /Ctrl2
Timm9
NaN
Npl
Pea15
Svip
Snapin
Eif3h
Tmsb10
NaN
Ppa1
Vamp2
Ptma
Bloc1s1
Hnrnpab
Nmt1
Rpsa
Vps29
Eny2
Mt3
Sncb
Nme2
Nipsnap1
Cutc
Calm1
NaN
Srsf4
Gale
Kctd12
Capn2
Brk1
Nrip2
Ddhd2
A1bg
Igsf11
Trove2
Otud7a
Ndufa4
Cox6a1
Asna1
Matn4
Nipsnap3b
Ccny
Mtr
M14-15, KI$^{\Delta CP}$ /Ctrl2
Ermn
Mbp
Bcas1
Dnajc5
Gdi1
Arpc4
Ca4
Igdcc4
Nhsl2
Kctd12
Cd9
Ankrd63
Adam11
Mgst3
Tardbp
Mpc1
Cd82
Lyrm9
Casp1
Omp
Tspan2
Chadl
Cyb5b
Pdcd2
Vps29
Camk2a
Dpysl2
Slc39a6
Zdhhc14
Hdhd2
Hnrnpab
NaN
Gkap1
Bok
Psmb2
Gbas
NaN
Hypk
Mfsd6
Ndufa4
Pfn2
Trappc2
Hbb-b1
Stat2
Rpl9
Cbln1
Actl6a
Dstn
M20-21, KI$^{\Delta CP}$ /Ctrl2
Anln
Cecr5
Lamc1
NaN
Rhog
Nid1
Plp1
Hebp1
Bcas1
Mag
Ttr
Endod1
Phldb1
Col4a2
Hpx
Atp1b3
Tmsb10
NaN
Tppp
Htra1
Wdfy1
Col4a1
Mrps18b
Ca14
Sncb
NaN
Cdc27
Enpp6
Vwa1
Hbb-b1
Col1a1
Mboat2
Cpm
Clic6
Rwdd2a
Tmem132e
Mug1
Rps15
Bysl
Yjefn3
Rnf13

Supplementary Figure 5. Volcano plots depicting differential expression of proteins in the hippocampus in $KI^{\Delta CP}$ mice compared to Ctrl1 and Crl2 at various ages (2-3, 5-6, 14-15, and 20-21 months). Shown are protein symbols, the written names are all the upregulated and downregulated proteins. Blue, significantly downregulated in $KI^{\Delta CP}$ compared to Ctrl, red, upregulated in $KI^{\Delta CP}$ compared to Ctrl.

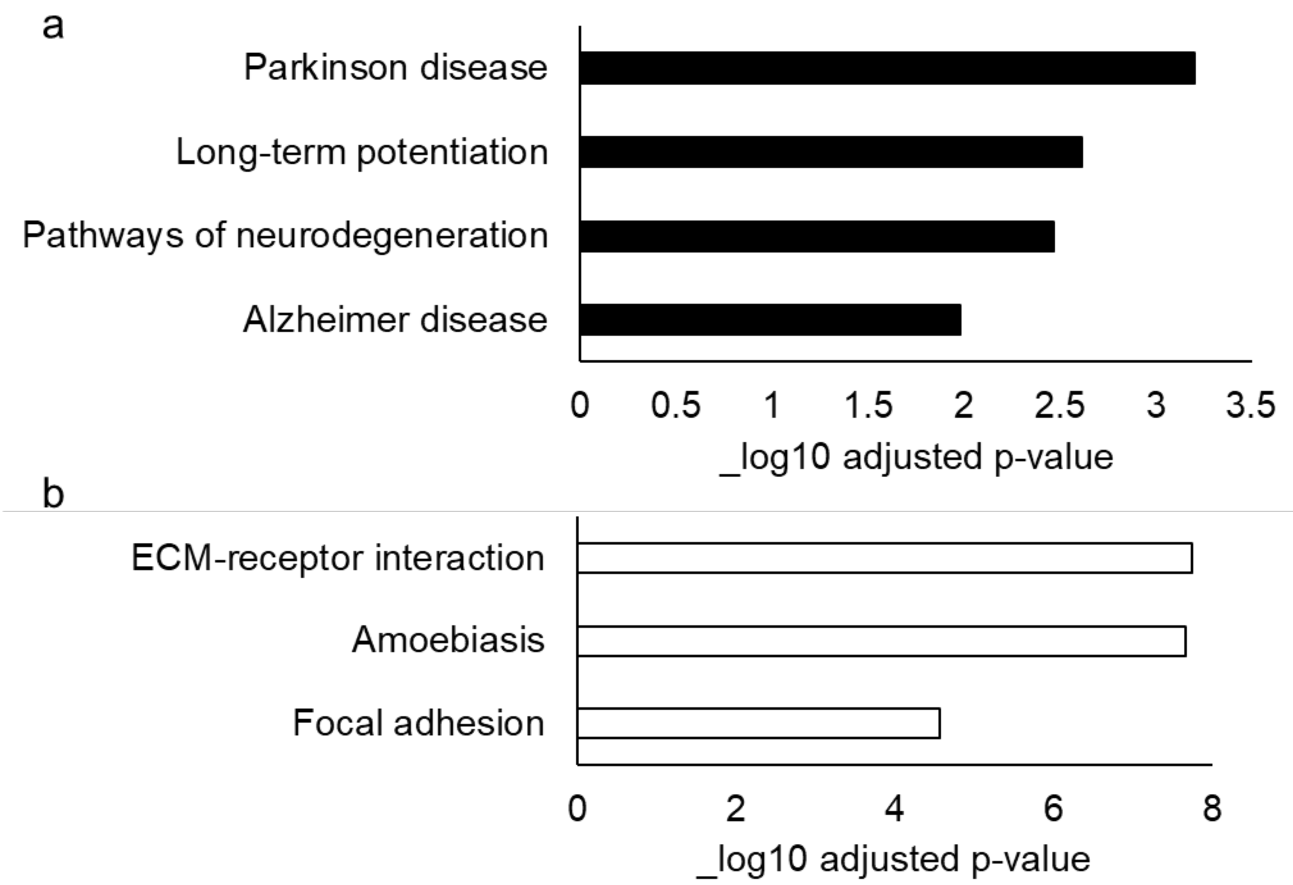

Supplementary Figure 6. KEGG pathway analysis of the proteins downregulated (a) and upregulated (b) (in all ages) in $KI^{\Delta CP}$ mice versus controls in the HC.
